# An “occlusive thrombosis-on-a-chip” microfluidic device for investigating the effect of anti-thrombotic drugs†

**DOI:** 10.1039/d1lc00347j

**Published:** 2021-08-12

**Authors:** Jess Berry, François J. Peaudecerf, Nicole A. Masters, Keith B. Neeves, Raymond E. Goldstein, Matthew T. Harper

**Affiliations:** Department of Pharmacology, University of Cambridge Tennis Court Road Cambridge CB2 1PD UK mth29@cam.ac.uk; Department of Civil, Environmental, and Geomatic Engineering, ETH Zürich 8093 Zürich Switzerland; Department of Bioengineering, Department of Pediatrics, Section of Hematology, Oncology, and Bone Marrow Transplant, Hemophilia and Thrombosis Center, University of Colorado Denver|Anschutz Medical Campus Aurora CO USA; Department of Applied Mathematics and Theoretical Physics, University of Cambridge UK

## Abstract

Cardiovascular disease remains one of the world's leading causes of death. Myocardial infarction (heart attack) is triggered by occlusion of coronary arteries by platelet-rich thrombi (clots). The development of new anti-platelet drugs to prevent myocardial infarction continues to be an active area of research and is dependent on accurately modelling the process of clot formation. Occlusive thrombi can be generated *in vivo* in a range of species, but these models are limited by variability and lack of relevance to human disease. Although *in vitro* models using human blood can overcome species-specific differences and improve translatability, many models do not generate occlusive thrombi. In those models that do achieve occlusion, time to occlusion is difficult to measure in an unbiased and objective manner. In this study we developed a simple and robust approach to determine occlusion time of a novel *in vitro* microfluidic assay. This highlighted the potential for occlusion to occur in thrombosis microfluidic devices through off-site coagulation, obscuring the effect of anti-platelet drugs. We therefore designed a novel occlusive thrombosis-on-a-chip microfluidic device that reliably generates occlusive thrombi at arterial shear rates by quenching downstream coagulation. We further validated our device and methods by using the approved anti-platelet drug, eptifibatide, recording a significant difference in the “time to occlude” in treated devices compared to control conditions. These results demonstrate that this device can be used to monitor the effect of antithrombotic drugs on time to occlude, and, for the first time, delivers this essential data in an unbiased and objective manner.

## Abstract 2

This occlusive thrombosis-on-a-chip microfluidic device can be used to test antithrombotic drugs. The device uses an on-chip chaotic mixer to introduce EDTA and quench downstream coagulation, essential for reliable determination of occlusion time.

## Graphical abstract

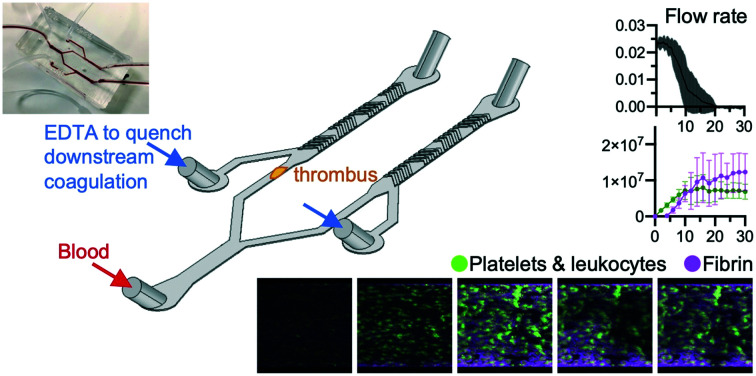

## Introduction

Cardiovascular disease (CVD) remains one of the world's biggest killers: in 2016 an estimated 17.9 million people died from CVD, accounting for 31% of global deaths. Of these CVD deaths, 85% were caused by myocardial infarction (heart attack) or stroke.^1^ The most common cause of myocardial infarction is arterial thrombosis at the site of atherosclerotic plaques in coronary arteries.^2^ Plaque rupture exposes fibrillar collagens and tissue factor, triggering platelet adhesion and rapid activation of circulating platelets.^3,4^ This leads to formation of a platelet-rich aggregate that is able to withstand the high shear rates within the arterial tree and occlude the flow of blood along the vessel.^5–7^

Compounds that effectively prevent arterial thrombosis are still the subject of intense research.^8–10^ All currently-used antithrombotic drugs are limited by increased bleeding risk, including potential fatal bleeds such as intracranial haemorrhage.^11–15^ Research efforts to discover improved medications are ongoing, but many compounds fail to translate from basic research to human clinical trials.^16–18^ To improve the success rate of drug discovery, better assays of occlusive thrombosis are needed.

Occlusive thrombi can be generated *in vivo* in a range of species, such as mice, rats, dogs and non-human primates.^19^ In these experiments, the time it takes for an artery to occlude after thrombosis is triggered is monitored.^20,21^ If pre-treatment with a compound delays or prevents occlusion, the compound is considered a potential treatment for the prevention of heart attacks and stroke that can be taken forward for further testing.^22,23^ However, results gained from these *in vivo* models are limited by variability and lack of relevance to human disease, and even promising compounds often fail to show an effect in clinical trials.^24,25^

*In vitro* models using human blood can overcome species-specific differences and improve translatability.^26–29^ In particular, *in vitro* models that incorporate blood flow, such as parallel-plate flow chambers, have increased our understanding of the underlying biology of thrombosis.^30–33^ Commonly, these flow models involve perfusing blood through a single channel coated in a thrombogenic substrate.^34^ With soft lithographical techniques becoming more widely used, an array of microfluidic thrombosis models has been published in recent years, incorporating pathologically important features such as stenosis.^29,35–38^ While these models mostly fail to produce occlusive thrombi, the ability of a thrombus to withstand the arterial flow of blood as it grows and the length of time it takes for the thrombus to cause full blockage of the artery are key determinants of mortality.^39,40^ Within many *in vitro* flow models, this occlusion process is unexaminable because blood is perfused into a single channel flow chamber at a set flow rate supplied by a syringe pump. As the thrombus grows within the channel, the increased resistance to fluid flow resulting from this growth causes a corresponding increase in pressure gradient across the top of the thrombus until its structural integrity is compromised; then the thrombus is dislodged, the channel cleared, and blood flow continues unimpeded.^35,41^ This process is unlike occlusive arterial thrombosis in the body, where thrombus growth in one artery diverts blood flow down different branches of the arterial tree.^42^ An alternative is to use a bifurcating device with two parallel arms, mimicking the branching arms of the arterial tree. The thrombogenic substrate is in one arm only. As the thrombosis grows on the substrate, the increased resistance diverts blood flow down the parallel arm, and occlusive thrombi can form on the substrate without a substantial increase in pressure being imposed upon them. This approach has been termed ‘pressure relief’.^35^ However, measuring time to occlude, as reported in *in vivo* models, is difficult in these devices. While channel occlusion has been reported in some of the published models,^43^ microscopy-based occlusion time measurements may be subject to experimental bias or inter-experimenter variation. Although in-line flow sensors can be used in microfluidic devices to detect cessation of flow in an unbiased manner, these devices are expensive and unsuitable for repeated blood work as blood is particularly challenging to remove during cleaning.

In this study we developed a simple and robust approach to determine occlusion time *in vitro*. This highlighted the potential for occlusion to occur downstream of platelet activation, and led us to design a novel thrombosis-on-a-chip microfluidic device that reliably generates occlusive thrombi. The time taken for occlusion to occur in a particular microfluidic device will be affected by channel geometry, flow rate and coagulant surfaces. Importantly, however, comparison of occlusion times generated with a defined assay can provide key data on efficacy of antithrombotic drugs. In this paper we describe a method to monitor the effect of antithrombotic drugs on time to occlude in an unbiased and objective manner. The assay developed generates data reflecting the efficacy of a known antithrombotic compound, eptifibatide, and exhibits sensitivity to changes in dose of the compound, providing strong evidence for the suitability of this assay in the identification of novel antithrombotic drugs.

## Materials & methods

### Device design and fabrication

To fabricate masters, SU8-2075 resin (A-Gas electronic materials) was spun onto a silicon wafer at 1500 rpm for 30s to produce a layer of 65–70 μm thickness. Coated wafers are soft-baked using a 65 °C heat pad for 5 minutes followed by a 95 °C oven for 20 minutes. Negative masks of the channel design were used to cure the resin by shining UV through the mask with a UV flood lamp (Oriel NUV illumination system; spectral band: 350–450 nm s; inverted configuration; power supply: Oriel Model 69910) for 3 × 11 s, with 15 s rest in between each exposure. The wafer was hard-baked using a 65 °C heat pad for 5 minutes and then transferred to a 95 °C oven for 11 minutes. Wafers requiring a second layer were re-spun, baked, exposed, and re-baked. Finished wafers were cleaned of uncured resin using a PGMEA bath for 9 minutes.

Devices were fabricated using poly(dimethylsiloxane) (SYLGARD-184 silicone elastomer kit; VWR chemicals), mixed at a ratio of 1 part curing agent:10 parts polymer. Mixed PDMS was transferred to 50 mL Falcon tubes and degassed using a 20s “pulse” spin by centrifuge (Thermofisher Megafuge 16R; pressing “pulse” results in an acceleration to 5000 rpm over 14 s followed by braking at maximum power when the button is released after 6 s). Degassed PDMS was poured on the master molds and baked for 45 minutes at 65 °C. After unmolding, exact chamber depth was confirmed by graticule measurement. A 2 μL spot of collagen I Horm suspension (Takeda; 0.1 mg mL^−1^) and tissue factor (Dade Innovin; 200 pM f.c.) was placed onto a cover slip and allowed to dry overnight at 4 °C. Prior to experiments, the spot was positioned centrally within one arm of the device. For initial single channel and pressure relief devices, the spot was placed centrally within the channel/upper arm of the device. For EDTA devices, careful placement of the PDMS ensured that the back edge of the patch was positioned just upstream from the point of EDTA entry into bloodstream. Following placement of the PDMS, the position of the spot within the channel was checked by eye and any devices where misplacement occurred were discarded. The PDMS was then vacuum-sealed to the coverslip: the device was designed to have a “vacuum chamber” surrounding all walls of the channel that is self-contained and remains separate from the main channel. By imparting a negative pressure into this chamber, the channel walls will be sucked down firmly onto the slide, sealing the device. We produced this vacuum using a very simple set-up requiring only a 10 mL syringe, tubing, and a lockable forceps. The 10 mL syringe was connected *via* a blunt needle to tubing leading into a pre-punched hole leading into the vacuum chamber. The plunger of the 10 mL syringe was then withdrawn to create a vacuum within the vacuum chamber, and with the plunger still withdrawn, the connecting tubing was clamped using lockable forceps and the forceps locked. Following this, the syringe and needle were removed. Devices could be “re-vacuumed” at any time by reattachment of the 10 mL syringe and withdrawal of the plunger, followed by unclamping and reclamping of the surgical forceps. To ensure functional blocking of the device, all tubing, syringes and needles were connected to sealed devices and the whole system filled with filtered bovine serum albumin (BSA; 2%; Sigma Aldrich, fatty-acid free) for at least 2 hours prior to use with whole blood.

### Blood collection and preparation

Blood was collected by venepuncture from healthy volunteers who had given informed, written consent and who had not taken medication for ten days. This procedure has been approved by the University of Cambridge Human Biology Research Ethics Committee. Following a discard draw of 5 mL, blood was drawn into 3.5 mL vacuettes (Greiner-Bio One) containing 3.2% sodium citrate. One vacuette was used per experiment: blood from the vacuette was emptied into a weigh boat and 3 mL of blood carefully drawn into a 5 mL syringe, stained with 3 μL DiOC_6_ (1 μM f.c.) and 15 μL purified human fibrinogen Alexa Fluor 546 conjugate (Thermo Fisher; 3.33 μg mL^−1^ f.c), then incubated at 37 °C for 10 minutes directly prior to use in the assay. In experiments using eptifibatide (Tocris, 10 μM f.c.), blood was pre-incubated with eptifibatide/DMSO control for 20 minutes prior to addition of DiOC_6_ and fibrinogen; as before, blood was then incubated for a further 10 minutes. Following incubation, the syringe containing blood was directly liquid-connected to the BSA-blocked 23ga needle inserted into silicone tubing and Tygon tubing leading to the inlet of the device (see Fig. S1† for detailed diagram). To allow coagulation, coagulation buffer (75 mM CaCl_2_ and 37.5 mM MgCl_2_) was added to the bloodstream *via* Y connector immediately prior to chamber entry at a rate of 1 : 9 buffer : blood. Residence time of buffer with blood was 2 minutes prior to chamber entry; adequate mixing had already been determined by preliminary experiments using fluorescein-stained coagulation buffer. To ensure coagulation buffer was being delivered to the device by the start of the experiment, BSA and coagulation buffer were run through the device for 5 minutes prior to connection of the blood-filled syringe, *i.e.*, these syringes were started during the final five minutes of incubation of the blood so all syringes had firm connections with their pumps by the start of the assay.

Blood is a sensitive and unstable medium with enormous potential for unwanted inter-experiment variation due to differences in set-up and handling.^44^ Experiments were designed in strict accordance with advisory papers published on this subject.^41,45–47^ Care was taken to replicate exactly both the experimental set-up, and the temperature changes and time periods between draw, incubation, and use of blood, on and between all experimental days. Experiments were run successively on each given day, which inevitably imposed sequential delays after the blood draw for each successive experiment. To overcome possible artifacts incurred by these delays, run order, ie, the order in which control and all variables were tested, was changed between experimental days until all experiments had been performed at each run order position, thus balancing any effects that may have been incurred across all experimental conditions.

### Shear rate calculations and addition of EDTA

All experiments used a shear rate of 1000 s^−1^ to mimic arteriolar shear. The flow rate *Q* in mL min^−1^ was calculated using the equation *γ̇* = 100*Q*/*H*^2^*W*) with *γ̇* shear rate in s^−1^, *H* and *W* the height and the width of the channel respectively, both in mm.^41^ For devices of depth 70 μm, *Q* = 0.0245 mL min^−1^. For devices where two arms run in parallel, the flow rate programmed into the pump was doubled with respect to the value obtained from the equation above.

EDTA-quenched devices incorporate an additional line of ethylenediaminetetraacetic acid solution (50 mM final concentration). To preserve osmolarity, quenching buffer was prepared using 1 : 10 0.5 M EDTA : HEPES buffer solution (HBS : HEPES 10 mM; NaCl 135 mM; KCl 3 mM; NaH_2_PO_4_ 0.34 mM; MgCl_2_·6H_2_0 1 mM). Chamber width doubles at the point of quenching buffer entry, so a flow rate of 0.0245 ml min^−1^ EDTA (*i.e.* matched to blood flow shear rate) was used to create a uniform shear profile in all areas of the device.

### Data collection and analysis

Thrombi growing within the chamber were imaged using a Zeiss LSM510 confocal microscope. A 10× objective was used to image a field of view of 921.36 × 719.81 μm. *Z*-Stacks were set up composed of 19 slices set at 5 μm intervals to cover the entire channel height (70 μm). Successive *z*-stacks were taken using 488 nm and 543 nm lasers. Each *z-*stack took 2 minutes to complete and was composed of alternating images in each channel – *i.e.*, the microscope was programmed to image each slice with first the 543 nm and then the 488 nm laser, and then shift the focus to image the next slice. Upon completion of each two minute stack, the next cycle was automatically begun, thus providing a time series of thrombi growth within distinct two minute windows. Data describing platelet and fibrin accumulation were generated by using the image analysis software Fiji.^48^ Each stack was collapsed into a maximum intensity projection, and the “measure” function of Fiji used to generate an “integrated density” summed pixel value for each projection. This was used as a measure of platelet and fibrin accumulation.

To generate data describing flow rate, the end of the waste tubing leading from the outlet downstream from the patch was placed into a collecting vessel placed on a balance. Recording was initiated when the first erythrocytes are seen entering the device, from which point balance data was recorded automatically every 5 s. Following the experiment, the initial weight of the tubing (*i.e.* the first recorded value) was subtracted from all values recorded during the experiment, and the data is converted from grams to milliliters using a conversion of 1 g = 1/1.06 mL. The time derivative of these data describes flow rate in mL min^−1^.

“Occlusion” was defined as a rate of less than 0.001 mL min^−1^, maintained for a period of 3 minutes or longer. “Occlusion time” defined by these criteria was identified for each repeat.

To determine the effects of quenching and treatment with 10 μM eptifibatide on platelet and fibrin accumulation, a two-way repeated measures ANOVA was performed comparing the integrated density values (see above) at 30 minutes, followed by Tukey's *post hoc* multiple comparisons test. To assess the effect of quenching and treatment on occlusion time, all channels that did not occlude were assigned a value of 40 minutes. These data were analysed using a one-way repeated measures nonparametric Friedman's test, followed by a *post hoc* Dunn's multiple comparisons test.

## Results

### Time to occlude can be measured in a “pressure relief” model of stable, occlusive thrombi

**Fig. 1:**
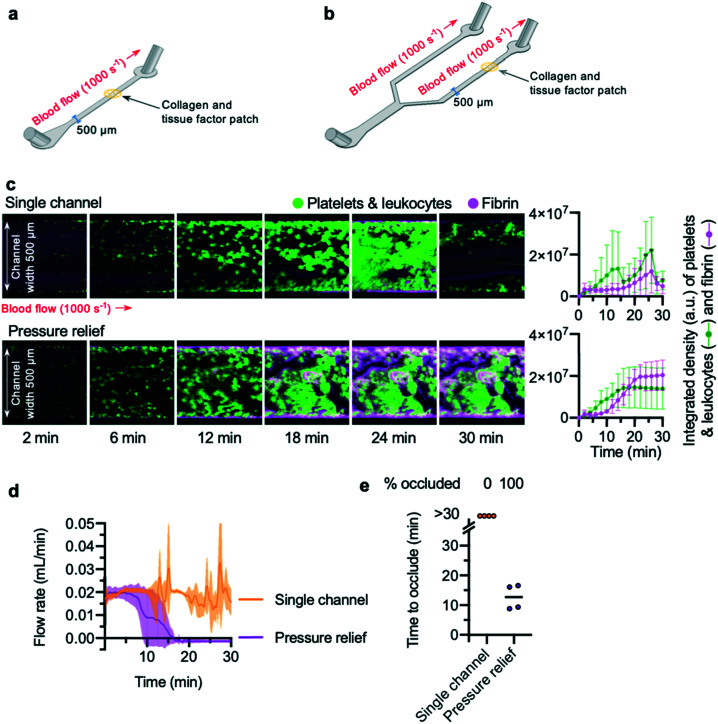
Occlusion time can be measured in pressure relief devices (a) schematic of a “single channel” device. The main channel of the device is of depth 65 μm and width 500 μm. Thrombosis was triggered by a patch of collagen and tissue factor spotted onto a glass slide and placed within the channel during device assembly. Whole blood was perfused across this patch at a shear rate of 1000 s^−1^ (b) schematic of a “pressure relief” device. (c) Confocal microscopy of thrombosis in single channel and pressure relief devices over a time-course of 2–30 minutes. Images shown are representational images, and blood in the two conditions is matched (same donor, same blood draw). The graphs to the right show mean ± standard deviation (SD) of the integrated density calculated from *n* = 4 donors, representing platelet and fibrin accumulation (see main text). Whole blood was stained with the lipid dye DiOC_6_ to visualise platelet aggregation, and spiked with fibrinogen-546 to visualise fibrin formation; areas of the device with enhanced accumulation of fibrin show an increased signal. Addition of a pressure relief arm to a traditional single channel microfluidic device is sufficient to allow occlusion. (d) Blood flow rate was calculated from the rate of increase of mass of fluid leaving the chamber, as described in the main text. The lines show mean with S.D. shown in the shaded area. (e) Pressure relief devices enable collection of data on “time to occlude” of channels, while single channels fail to occlude. Time to occlude was calculated from blood flow data, as described in the main text.

Previous studies^35,43^ have reported that addition of a bifurcation into a channel allows occlusive thrombi to form. To confirm these results, a “single channel” microfluidic device and a “pressure relief” microfluidic device were designed and fabricated. The “single channel” microfluidic device was designed with a single inlet and outlet, connected by a straight channel (Fig. 1a). In the “pressure relief” microfluidic device, a single inlet connects *via* a branching design to two outlets (Fig. 1b). To mimic plaque rupture within the devices, a patch of collagen and tissue factor was spotted onto a glass slide and positioned within one channel of the device (see the Methods section for more detail).

Citrate-anticoagulated whole blood, recalcified by a buffer added to the blood *via* a Y-connector just prior to chamber entry, was perfused into the device at a flow rate calculated to generate an initial shear rate across the collagen and tissue factor patch that mimics arteriolar conditions (1000 s^−1^). To visualise thrombus formation, platelets (and leukocytes) were labelled with the lipid dye, DiOC_6_. Fibrin was detected using fibrinogen Alexa-546 conjugate. *Z*-Stacks of DiOC_6_ and Alexa-546 fluorescence of the growing thrombus on the collagen and tissue factor patch were acquired in two-minute intervals.

Platelet aggregation and fibrin formation within single channel devices was highly variable, and often resulted in embolism: the growing clot detached from the collagen and tissue factor patch (Fig. 1c; Video S1†). By contrast, platelet aggregation and fibrin formation within pressure relief devices took place at a steady rate (Fig. 1c; Video S2†), producing thrombi that were stable for the duration of the experiment (30 min).

To quantitatively determine occlusion time in a low-cost and easily reproducible manner, we weighed the blood efflux leaving the waste tubing downstream from the collagen and tissue factor patch. The weight recorded by the balance was converted into volume of blood, and the rate of change of blood volume used to calculate blood flow rate in mL min^−1^ (Fig. 1d). This approach avoids the need to place an in-line flow sensor in direct contact with blood.

The flow rate in single channel devices reflected the unpredictable accumulation of platelets and fibrin in these experiments: flow did not cease and remained unstable, with large increases and decreases seen as embolism events occurred. In contrast, the flow rate in pressure relief devices decreased over time, leading to cessation of flow (Fig. 1d). These data were used to determine the “time to occlude” for each experiment. “Occlusion” was defined as a flow rate of 0.001 mL min^−1^ or less, maintained for three minutes or longer. In all experiments using a single channel device, these criteria were never met, whereas all pressure relief devices occluded within 20 minutes (Fig. 1e).

Of note, we observed substantial permeation of erythrocytes and leukocytes into the thrombus throughout the entire duration of the pressure relief experiments. While post-analysis of the experimental data from the balance objectively confirmed that bulk flow had ceased, the continued permeation of erythrocytes and leukocytes would have made it difficult to use microscopy alone to determine occlusion in an objective and repeatable manner. These observations underscore the importance of using an unbiased measure of flow rate when assessing medications.

### ‘Off-site’ coagulation can generate erroneous occlusion times

**Fig. 2:**
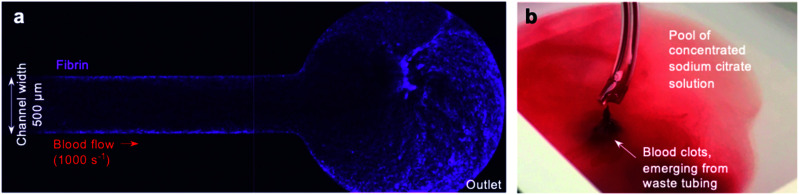
Off-site coagulation downstream from the collagen and tissue factor patch (a) fibrin formation (magenta) was observed spreading along the channel in some devices, including at the outlet. The image shown is a representative image of a pressure relief device, and depicts the outlet situated downstream from the collagen and tissue factor patch. The image was taken 30 minutes after initiation of the experiment using a 4× objective. (b) Clots were observed emerging from the end of the waste tubing. Collecting into a vessel of concentrated sodium citrate was not sufficient to prevent this.

Despite these promising initial results, we observed that some devices exhibited fibrin formation spreading along the arm of the device downstream from the collagen and tissue factor patch (Fig. 2a), and that clots often emerged from the waste tube into the waste collection vessel. Collecting this waste from the chamber into sodium citrate solution did not prevent occurrence of clots in the waste (Fig. 2b). These observations raised queries about the nature of the occlusion within our pressure relief device.

We hypothesised that even a small number of platelets activated at the collagen and tissue factor patch may quickly propagate into widespread activation of the coagulation cascade and fibrin formation downstream from the patch. Alternatively, coagulation initiated at the patch may propagate downstream, as previously seen in a larger parallel-plate-based device.^49^

Whilst the coagulation cascade is known to be a factor in arterial thrombosis, *in vivo* it is understood to be a secondary event that follows a platelet-driven occlusion,^50^ with thrombi limited to the point of vessel injury or plaque rupture by the remaining intact vasculature.^51^ Conversely, in pressure relief microfluidic devices, unregulated coagulation could become a feed-forward event: coagulation triggered in one arm of the device may increase the viscosity of blood within this arm, leading to a diversion of flow into the other arm. The reduced flow rate in the first arm would promote further coagulation, quickly blocking one arm of the device and the waste tubing downstream. The feed-forward nature of this coagulation-driven blockage could be relatively unrelated to the level of platelet activation and could occur at sites away from the collagen and tissue factor patch.

This off-site coagulation could affect results by promoting occlusion. Instead, if our device was to provide an accurate model of *in vivo* arterial thrombosis, occlusion must be driven by platelet activation on the collagen and tissue factor patch alone, with the thrombus and subsequent coagulation spatially limited to this area of the device.

### Development of an ethylenediaminetetraacetic acid (EDTA)-quenched device to prevent coagulation downstream of prothrombotic substrates

**Fig. 3:**
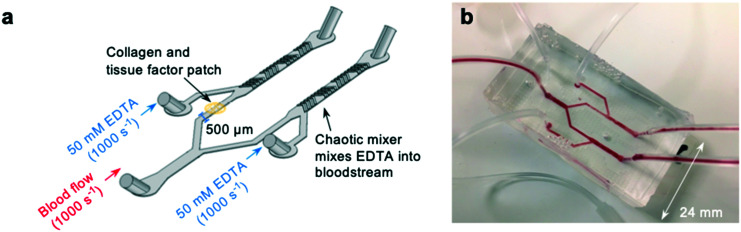
Development of a novel “EDTA-quenched” device incorporating a chaotic mixer (a) schematic of the EDTA-quenched device design. A stream of high-concentration (50 mM) EDTA is introduced just after the collagen and tissue factor patch and mixed into the bloodstream using a chaotic mixer. (b) A photograph of the fabricated device.

To investigate whether off-site coagulation could be affecting occlusion in pressure relief devices, we developed an EDTA-quenched microfluidic device (Fig. 3a and b). EDTA is an effective chelator of calcium and magnesium that inhibits platelet and leukocyte activation and prevents coagulation. We hypothesised that introduction of EDTA downstream from the patch of collagen and tissue factor would neutralise off-site coagulation and prevent downstream occlusion.

The device incorporates two additional inlets that supply high-concentration EDTA solution immediately downstream from the collagen and tissue factor patch. Mixing of fluid flow within microfluidic devices is known to be minimal within microfluidic channels due to low Reynolds numbers and the resulting undisturbed laminar flow profiles.^52–55^ To ensure the EDTA solution would mix into the blood stream, a chaotic mixer was incorporated into the device design.^56^ To form the mixing section of the device, a second layer, also 65 μm deep, was fabricated on top of the first during fabrication of the molds (see methods section for a detailed account of this process). This layer consisted of asymmetric bands, each 50 μm wide, repeated along the length of the outlet downstream from the inlet providing EDTA-solution (Fig. S2a†). Thus, when cast, these bands would be present in the device as an extruding pattern in the roof of the device, and function to create static mixing between the blood flow and the EDTA-solution.

To check that the mixer was successful, whole human blood was stained with rhodamine, and EDTA-solution stained with fluorescein (Fig. S2b†) and the mixing of the fluorescein across the channel was plotted (Fig. S2c†). The chaotic mixer successfully dispersed the EDTA solution across the whole width of the channel by the point at which blood reaches the outlet.

### The EDTA-quenched device allows assessment of the efficacy of antithrombotic compounds in preventing occlusive thrombus formation

**Fig. 4:**
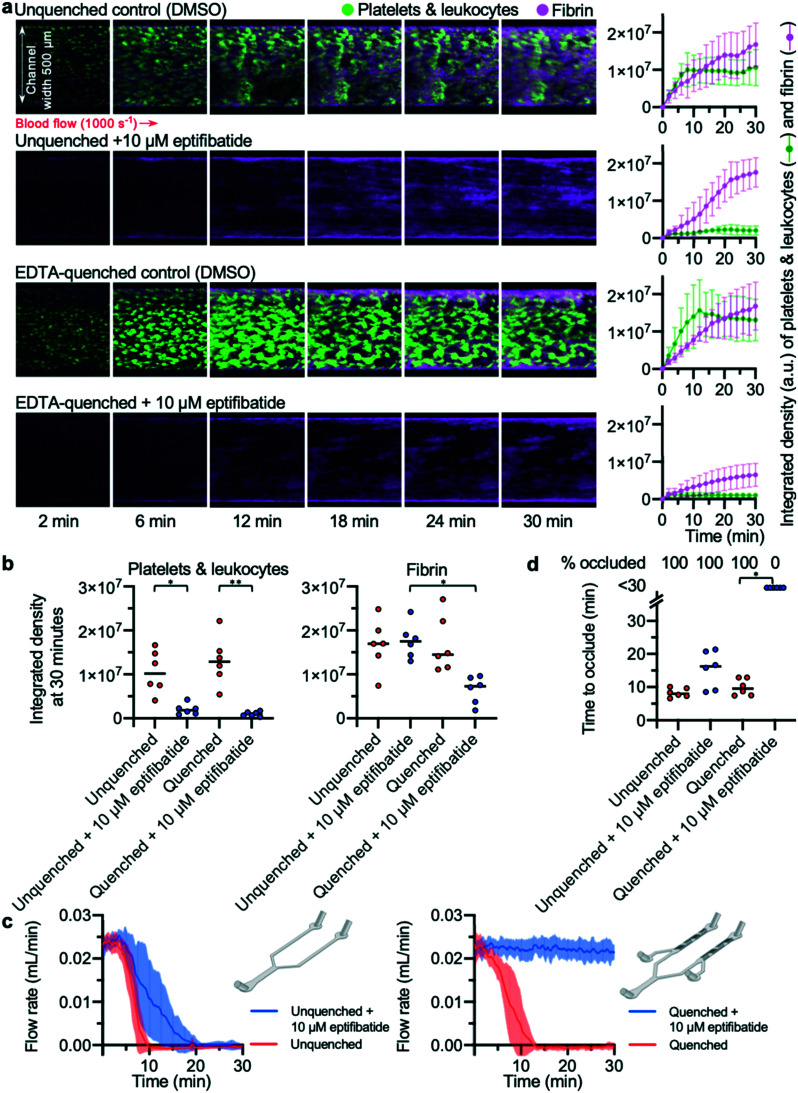
EDTA-quenching is necessary to observe the anti-thrombotic effect of eptifibatide (a) platelets (and leukocytes) and fibrin were visualised using confocal microscopy over a 30 min time-course. The images are representative of *n* = 6 donors. Mean platelet and fibrin accumulation ± S.D. is shown in the graphs (*n* = 6). (b) The platelet and fibrin integrated density values at 30 min. Treatment with eptifibatide had a significant effect on platelet accumulation in both devices (unquenched *P* = 0.0128(*); EDTA-quenched *P* = 0.0027(**)). Treating blood with eptifibatide had a significantly different effect on fibrin accumulation depending on which device was used (*P* = 0.0456(*)). (c) Mean blood flow rate in each condition with S.D. shown in the shaded area (*n* = 6). (d) Time to occlude for each experiment is shown in the graph. Treating with eptifibatide had a significant effect when using EDTA-quenched devices (*P* = 0.0219(*)) but no significant effect in unquenched devices.

To validate the EDTA-quenched device, we compared the time to occlude reported in this device to the unquenched pressure relief device used in previous experiments. Platelet deposition and fibrin formation in both unquenched and EDTA-quenched controls were similar; in both devices, thrombi steadily increased in size over time and formed stable platelet and fibrin-rich structures (Fig. 4a). To assess whether off-site coagulation was affecting the occlusion in unquenched pressure relief devices, we used blood treated with 10 μM eptifibatide, a potent inhibitor of platelet integrin α_IIb_β_3_. Eptifibatide almost entirely abolished platelet aggregation in both devices (Fig. 4a). In EDTA-quenched devices, fibrin formation was also substantially reduced when eptifibatide-treated blood was used. By contrast, in the eptifibatide-treated unquenched device, fibrin accumulated at a rate similar to that of the untreated control. The integrated density values for platelets and fibrin at 30 minutes provide a comparable quantification of thrombus growth in the different conditions tested (Fig. 4b). No significant difference was found between the untreated control experiments performed in either an unquenched or EDTA-quenched device. As expected, eptifibatide significantly reduced platelet accumulation in both devices when compared to their respective control experiment. In contrast, analysis of fibrin accumulation using a two-way ANOVA found that EDTA-quenching and eptifibatide treatment showed significant interaction, with significantly lower fibrin accumulation in quenched devices treated with eptifibatide compared to unquenched devices also treated with eptifibatide.

Comparison of flow rates from these devices revealed the importance of abolishing downstream coagulation when assessing the efficacy of antithrombotic compounds (Fig. 4c). While thrombus formation in both unquenched and EDTA-quenched control experiments developed at a similar pace, eptifibatide had different apparent effects on the occlusion time, depending on which device was used (Fig. 4d). Occlusion occurred in all unquenched devices treated with eptifibatide, with only a minor delay in occlusion time compared to matched control (Fig. 4d), despite the channels of these devices remaining almost entirely clear of thrombi at the collagen and tissue factor patch (Fig. 4a). In contrast, when eptifibatide was tested using the EDTA-quenched device, flow rate did not decrease for the duration of the experiment (Fig. 4c), and none of the devices occluded (Fig. 4d). Only the EDTA-quenched device was able to reveal the efficacy of eptifibatide in preventing occlusive thrombosis.

### The EDTA-quenched device displays concentration-dependent inhibition of occlusive thrombosis by eptifibatide

**Fig. 5:**
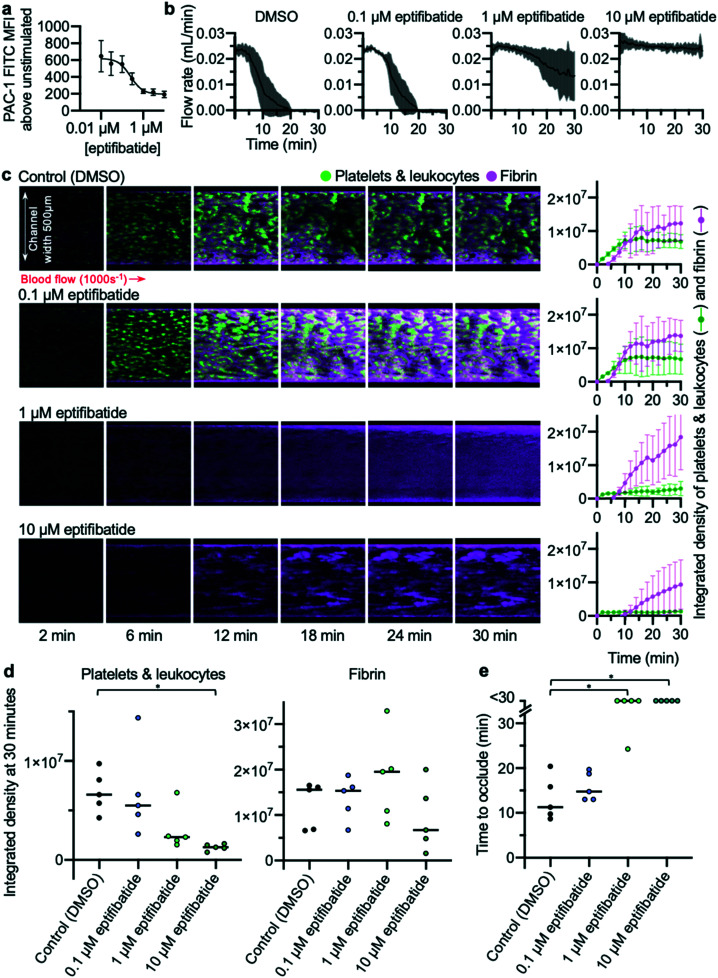
Concentration-dependent inhibition of occlusive thrombosis by eptifibatide (a) whole blood flow cytometry shows concentration-dependent inhibition by eptifibatide of antibody PAC-1 binding to activated integrin α_IIb_β_3_ (*n* = 5). (b) Changes in the concentration of eptifibatide were reflected in flow rate data collected from the developed microfluidic assay. (c) Platelets (and leukocytes) and fibrin were visualised using confocal microscopy over a 30 min time-course. The images are representative of *n* = 5 donors (matched with flow cytometry data). Mean platelet and fibrin accumulation ± S.D. is shown in the graphs (*n* = 5). (d) The platelet and fibrin integrated density values at 30 min. Treatment with 10 μM eptifibatide had a significant effect on platelet accumulation (*P* = 0.02(*)). (e) Time to occlude for each experiment is shown in the graph. Treating with 10 μM and 1 μM eptifibatide had a significant effect on the time to occlude compared to control (10 μM eptifibatide *P* = 0.0197(*); 1 μM eptifibatide *P* = 0.0423(*)).

The effects of different concentrations of eptifibatide were tested using the EDTA-quenched device. Whole blood flow cytometry experiments were performed concurrently in which ability of PAC-1 antibody to bind to activated αII_b_β_3_ was measured (Fig. 5a). Concentrations of 10, 1 and 0.1 μM eptifibatide were tested using the EDTA-quenched device. Flow rates generated in these experiments reflected the changes in concentration of eptifibatide pre-incubated with the blood (Fig. 5b). The time course of platelet accumulation was also inhibited by eptifibatide in a concentration-dependent manner (Fig. 5c), with marginal effects seen when blood was incubated with 0.1 μM eptifibatide, whereas, as before, platelet aggregation was significantly inhibited by incubation with 10 μM eptifibatide. Data collected on occlusion time (Fig. 5d) allowed data on flow rate to be quantified at the 30 minute end-point of the experiment, and both 1 μM eptifibatide and 10 μM eptifibatide were found to significantly delay occlusion time beyond 30 minutes.

Taken together, these data provide a detailed picture of the efficacy of differing concentrations of eptifibatide in preventing platelet aggregation, and prevention of occlusion. Data collected using the flow assay mirrored those gathered using flow cytometry where the effects of eptifibatide on its target were measured directly, further supporting our approach. The assay and imaging methods developed were able to differentiate between increasing concentrations of eptifibatide, and the data collected provides strong evidence that the assay is suitable as a screening tool for anti-thrombotic medications.

## Discussion

In this study we present an *in vitro* occlusive thrombosis-on-a-chip microfluidic device that allows measurement of time to occlude and can be used to study the ability of compounds to prevent occlusive thrombi. Occlusion time is a commonly reported parameter in animal models of thrombosis, such as the murine carotid artery ferric chloride-induced injury model. However, recent studies have questioned the physiological relevance of this model into question, with ferric chloride (FeCl_3_) shown to have non-specific effects on multiple isolated blood cell-types,^27^ and electron microscopy images of the initial stages of thrombosis induced in the murine carotid artery by FeCl_3_ have revealed that the thrombosis is triggered by erythrocytes.^57^ These studies make a powerful case for the induction of thrombosis by FeCl_3_ to be viewed as a response to chemical injury, not a specific biological response that mimics arterial thrombosis. *In vitro* models provide precise control over the prothrombotic trigger in the model, and also can use human blood, making the data more translatable to human disease. However, time to occlusion has been challenging to assess from *in vitro* models.

In this study, we replicate the work of other groups in showing that addition of a bifurcation into a simple microfluidic channel is sufficient for occlusion to occur^35,43^ despite constant flow rate into the device. Alternative approaches exist that allow occlusion to occur by producing a constant pressure drop across the system: constant pressure pumps monitor the pressure of a system and adjust pressure accordingly; hydraulic towers at the inlet produce a pressure drop across the system from inlet to outlet; and negative pressures applied to outlets *via* vacuum pumps generate pressure drop from inlets at atmospheric pressure.^36,58–60^ Depending on the method used to generate constant pressure at the start of the experiment, as the thrombus builds, the pressure, flow rate, and shear rate within the device may vary.^35^ This is similar to conditions found *in vivo*, where a thrombus growing within the branching structure of an artery will affect flow of blood along that arm of the vessel.^61^

Here we present a simple solution to measure the ‘time to occlude’ of *in vitro* channels. By weighing the fluid leaving the device tubing downstream from the site of activation, the flow rate can be measured without requiring in-line flow sensors, which can be difficult to clean if they are to be re-used. By contrast, we offer a low-cost, easily implemented solution utilising a balance, similar to approaches described elsewhere.^62–64^ The quantitative data provided by this method allows unbiased assessment of the efficacy of compounds to prevent or delay occlusion. This represents an improvement upon the most common alternative, in which experimenters estimate time to occlude by microscopic observation of flow cessation. This approach can be subjective and prone to inter-experimenter variation, particularly since some permeation of erythrocytes through the clot can be observed after occlusion has occurred, as reported previously.^65^

Our time to occlude data revealed that off-site (downstream) coagulation in *in vitro* arterial thrombosis models is a major concern and that this coagulation must be prevented in order for the data describing “time to occlude” to be biologically accurate. This is revealed by comparing the effect of eptifibatide, an integrin α_IIb_β_3_ inhibitor, on thrombosis and time to occlude in the devices with EDTA-quenching to prevent off-site coagulation and in those without EDTA quenching. As expected, eptifibatide abolished platelet aggregation, with only single platelet adhesion observed. This inhibition was seen in both EDTA-quenched and unquenched devices. However, in the unquenched eptifibatide-treated device, despite the lack of thrombus formation, flow rate slowed and ceased with only a marginal delay in comparison to the control without eptifibatide. This demonstrates that downstream coagulation in these devices was giving a ‘time to occlusion’ that did not correlate with the extent of thrombosis at the collagen and tissue factor site. In contrast, in our EDTA-quenched eptifibatide-treated device we observed undiminished flow rate throughout the duration of the experiment (30 min), consistent with the lack of thrombus formation on the collagen and tissue factor patch. These results show that off-site coagulation within *in vitro* arterial thrombosis models masks the effects of antithrombotic drugs but can be prevented by our EDTA-quenched device.

Off-site coagulation also alters the process of thrombosis, as seen by the difference in fibrin formation after eptifibatide treatment in EDTA-quenched and -unquenched devices. Initially, fibrin accumulated on the collagen and tissue factor patch at a similar rate in the two devices. However, as coagulation increased downstream in the unquenched devices, flow began to slow, thus allowing the rate of fibrin accumulation on the patch to increase. By 30 minutes, the fibrin accumulation in unquenched devices was no different between eptifibatide-treatment and control. In contrast, when blood flow was maintained in EDTA-quenched devices, fibrin accumulation was significantly inhibited by eptifibatide, revealing the role of activated platelet aggregates in accelerating thrombin generation at the site of injury.^66,67^

The efficacy of eptifibatide in preventing occlusion is plain to see when assessing the data generated from the EDTA-quenched device. It is alarming how different the conclusions drawn about the efficacy of eptifibatide would be, were only the results from the pressure relief device assessed. In these unquenched devices, it appears that eptifibatide has no effect on occlusion. Despite the almost complete lack of platelet aggregation in these experiments, fibrin builds within the channel and leads to a cessation of flow. These results could be believed, were it not for the stark contrast with the EDTA-quenched device: when downstream activity is quenched by EDTA, the fibrin at the site of the collagen and tissue factor patch is nowhere near sufficient to even slow flow rate, let alone occlude the channel.

Quenching of downstream coagulation is therefore necessary to model thrombosis at a plaque rupture. We suggest that quenching mimics the biological action of endothelial cells, which neutralise downstream coagulation,^68^ spatially limiting thrombosis and preventing it from spreading along the entire arterial tree.^69–71^ An alternative to our quenching design might be to endothelialise the device, although even when the device channels are endothelialised, coagulation may still occur in the waste tubing and impact the flow rate. Additionally, the channel design would need to be modified further to allow exposure of collagen and tissue factor. These prothrombotic triggers were chosen to mimic a ruptured atherosclerotic plaque.^3,72,73^ In contrast, thrombosis in many endothelialised devices is triggered by activated (but intact) endothelial cells,^43,74–76^ modelling a different disease state and exposing a different repertoire of pro-thrombotic signals.

In our assay we have used collagen and tissue factor to activate platelets and trigger thrombosis, mimicking the exposure of these substrates when an atherosclerotic plaque ruptures. In the body, however, atherosclerotic plaque development is naturally accompanied by progressive stenosis (narrowing) of the vessel,^2^ which is also known to be an important trigger of thrombosis. A number of microfluidic systems have been developed that explore the thrombotic effect of stenoses; results from these assays have uncovered the importance in both the percentage occlusion incurred by the stenosis and the gradient of the post-stenosis deceleration zone.^77,78^ These studies assess the effect of stenosis alone. A consideration for the future would be to develop a model of arterial thrombosis that combines the effect of stenosis with activating substrates, thus recreating both the physical and biological triggers of arterial thrombosis in one assay. Considerable barriers remain to these developments, however. The top-down design of soft lithographical models means that stenoses incorporated into channel geometries protrude from the side of the channel, not from the floor.^79–81^ Collagen and tissue factor can only be introduced as a concurrent activator by means of patterning them onto the floor of the device next to the stenosis that protrudes from the channel wall.^79^ Some groups have attempted to overcome the limitations of traditional soft lithographical designs by creating round PDMS channels^26^ or PDMS models of stenotic vessels excised from diseased patients.^82^ These assays more accurately recreate the geometry of blood vessels, but once again, platelet activation is induced by stenosis alone, as collagen and tissue factor cannot be patterned within these solid PDMS designs. Despite these difficulties, the incorporation of both elements into a single device represents a promising avenue of investigation.

## Conclusions

We have developed a thrombosis-on-a-chip device that allows biologically relevant occlusive thrombi to form at arterial shear rates, which can be used to assess the effect of anti-thrombotic compounds on the time to occlude. Key features of our device are the branching design of the channel, incorporation of an EDTA-quenching buffer downstream of the thrombotic site, and a simple but robust method to measure time to occlude. Importantly, the downstream EDTA-quenching of coagulation is necessary to see the effect of a potent anti-platelet drug on time to occlusion, and demonstrated that off-site coagulation in *in vitro* devices can distort the apparent biological response. Our device could be readily modified to include further disease relevant features, such as different pro-thrombotic substrates or stenotic channel geometries. In time, we hope that such devices will replace many of the *in vivo* experiments currently performed in thrombosis research.

## Conflicts of interest

There are no conflicts to declare.

## Supplementary Material

LC-021-D1LC00347J-s001

LC-021-D1LC00347J-s002

LC-021-D1LC00347J-s003
