# Supplementary material for: An “occlusive thrombosis-on-a-chip” microfluidic device for investigating the effect of anti-thrombotic drugs
Source: Lab Chip. 2021 Aug 12;21(21):4104–17. doi: 10.1039/d1lc00347j (PMC8547327; doi:10.1039/d1lc00347j)
Supplement: LC-021-D1LC00347J-s001 [file LC-021-D1LC00347J-s001.pdf]

Electronic Supplementary Material (ESI) for Lab on a Chip.
This journal is © The Royal Society of Chemistry 2021

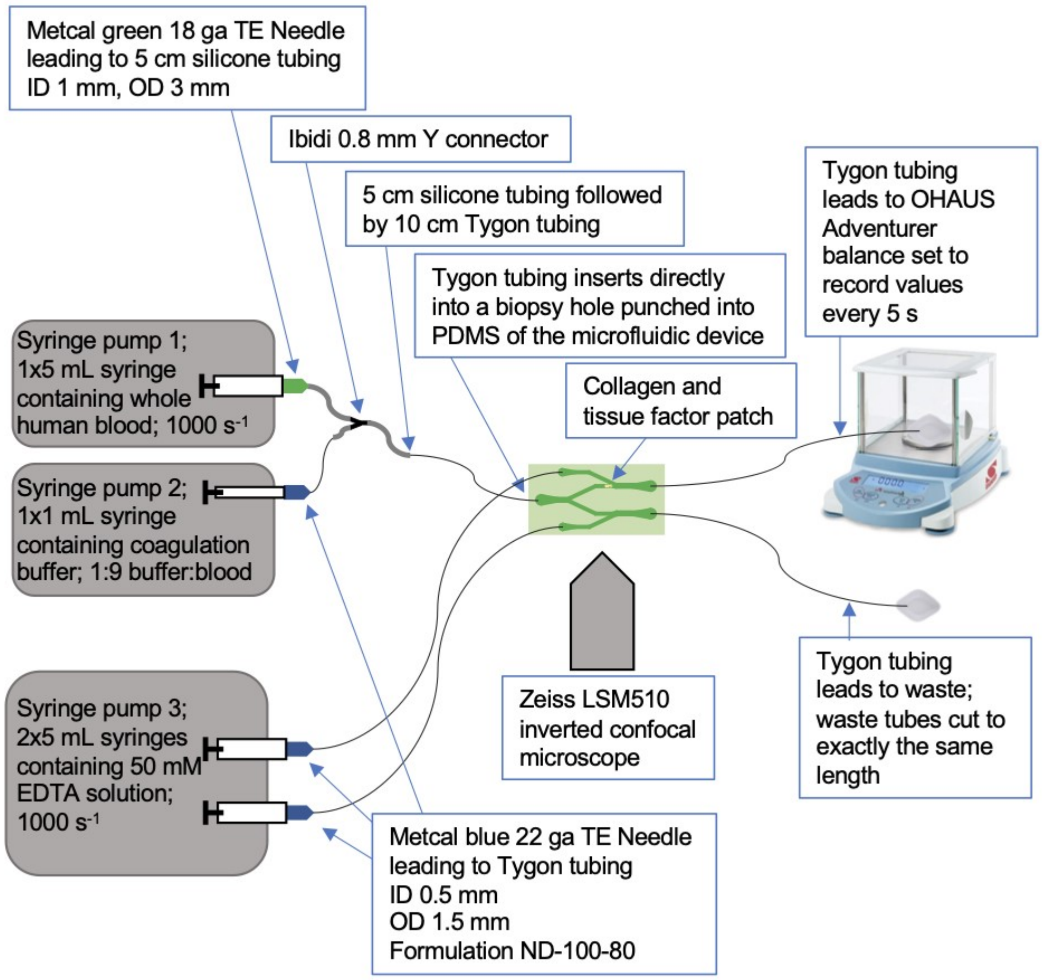

Supplementary fig. 1 Schematic of experimental set-up

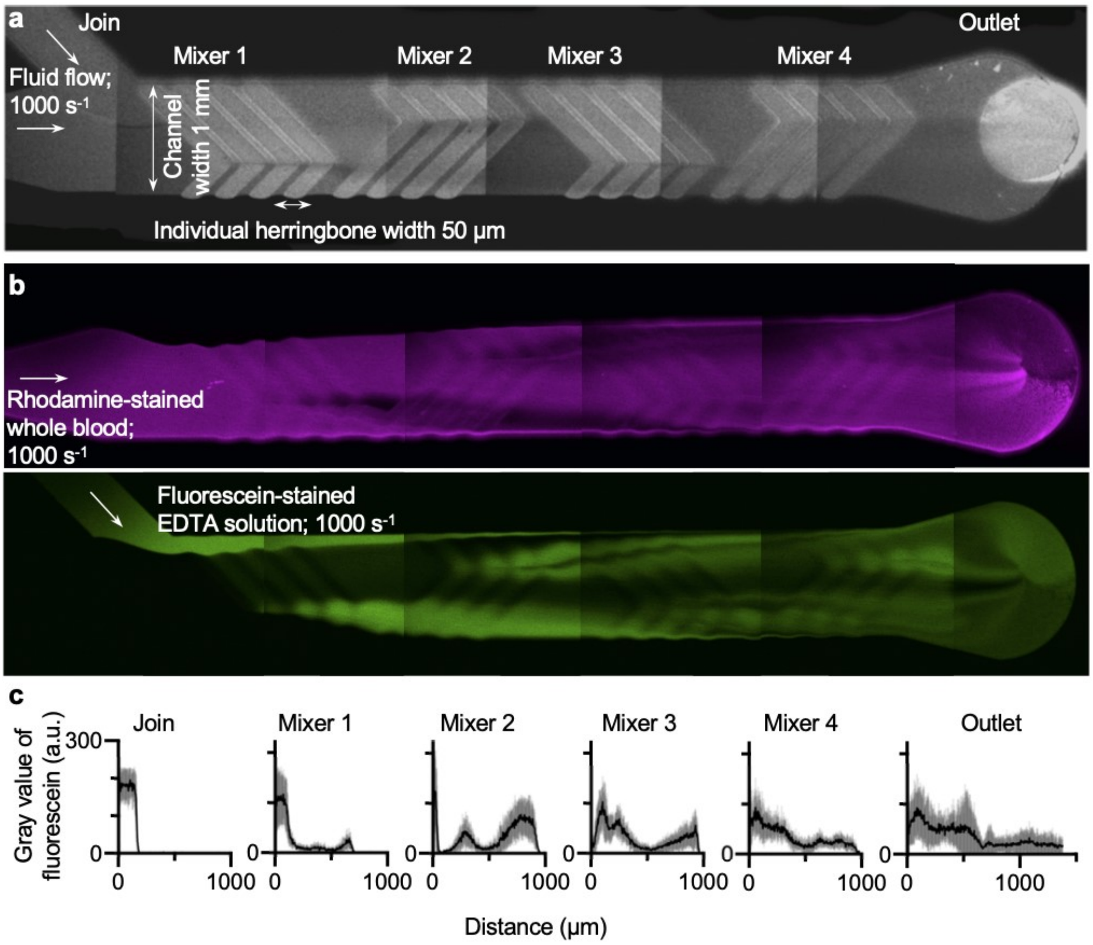

Supplementary fig. 2 Details of the chaotic mixer (a) The chaotic mixer consists of 50 µm bands repeated across the top of the channel. **(b)** The chaotic mixer is sufficient to mix the EDTA stream across the width of the channel. Whole blood stained with rhodamine (magenta) was flowed into the device from the main inlet, and EDTA-solution stained with fluorescein (green) was flowed into the device from the EDTA inlet. By the end of the channel, fluorescein was present across the width of the outlet. **(c)** Plots of the pixel values confirm the presence of fluorescein across the width of the channel (n=3).
